# Prognosis comparison between small cell carcinoma of ovary and high-grade serous ovarian cancer: A retrospective observational cohort study

**DOI:** 10.3389/fendo.2023.1103429

**Published:** 2023-01-18

**Authors:** Dongzhi Hu, Dongdong Ma, Zi-jian Zhang, Yongjing Zhang, Kangni Huang, Xiaoxue Li

**Affiliations:** ^1^ Department of Obstetrics and Gynecology, Yiyang Central Hospital, Yiyang, China; ^2^ Department of Pharmacy, Guangxi University of Science and Technology, Liuzhou, China; ^3^ Department of Obstetrics and Gynecology, The Second Xiangya Hospital of Central South University, Changsha, China

**Keywords:** small cell carcinoma of ovary, high-grade serous ovarian cancer, overall survival, cancer-specific survival, SEER database, Propensity score matching, antigen 125

## Abstract

**Background:**

Small cell carcinoma of ovary (SCCO) is a rare and aggressive cancer primarily reported in the form of case reports. Due to limited epidemiological and prognostic analyses based on large populations, SCCO has varied considerably without prognostic models and a recognized first-line treatment strategy. The study aimed to compare the clinical characteristics, treatment methods, and prognosis of SCCO and high-grade serous ovarian cancer (HGSOC), the most prevalent subtype of ovarian cancer, in a large sample and develop a predictive model for these two subtypes.

**Methods:**

Data from the Surveillance, Epidemiology, and End Results program were analyzed for patients with SCCO or HGSOC from 2000 to 2017. Clinical, demographic, and treatment characteristics were compared between the two groups. Propensity-score matching, Cox risk regression analysis, and Kaplan-Meier survival curves were used to assess the data. Finally, a nomogram was developed to predict the patient survival time.

**Results:**

A total of 32,185 women, including 31,979 (99.4%) diagnosed with HGSOC and 206 (0.6%) diagnosed with SCCO, were identified. Age ≤ 51 years, single, median house income less than $70,000, early stage, and unilateral disease were more common characteristics of patients with SCCO than those with HGSOC. Patients with SCCO were more likely to receive radiotherapy (6.8% vs. 0.8%, p <0.001) and have tumors ≥ 141 mm (38.3% vs. 9.7%, p <0.001) than patients with HGSOC. The independent risk factors for SCCO patients included older age at diagnosis, advanced stage, surgery, radiotherapy, chemotherapy, larger tumor size, and bilateral tumor. Overall and cancer-specific survival rates were significantly lower for SCCO than more malignant HGSOC. Prognostic models and nomograms had been constructed to predict the individual survival rates of patients with SCCO and HGSOC.

**Conclusion:**

Patients with SCCO presented with the early-stage disease more frequently than patients with HGSOC and had decreased overall and cancer-specific survival rates.

## 1 Introduction

Ovarian cancer remains one of the most lethal gynecological malignancies, accounting for 2.5% of all female malignancies (1, 2). It is estimated that 19,880 new cases of ovarian cancer would be diagnosed, with 12,810 deaths in the United States in 2022 (3). Ovarian cancer is a heterogeneous disease categorized as histological subtypes with different epidemiology, treatment strategy, and prognosis (4–6). About 90% of ovarian tumors are believed to originate from the epithelium cells histologically (7), with high-grade serous ovarian cancer (HGSOC) accounting for the majority of ovarian epithelial tumors (8). Standard treatments for newly diagnosed HGSOC include cytoreductive surgery and platinum-based chemotherapy, with most patients becoming platinum resistant. Hence, HGSOC is more aggressive (5-year cause-specific survival is 43%) than most of the other subtypes of ovarian cancer (5-year cause-specific survival ranging from 66-82%) (9).

Small cell carcinoma of ovary (SCCO) is a rare and aggressive cancer, primarily affecting adolescents and young women, usually accompanied with the hypercalcemic type (SCCOHT) and SMARCA4 mutation (10). SCCO has primarily been reported as case reports on PubMed, accounting for less than 0.01% of all ovarian malignancies. The diagnosis, genetic counseling, epidemiology, and treatment strategies of SCCO remain controversial due to the lack of clinical data on this rare malignancy (5, 11). SCCO is currently grouped into various neoplasms in the World Health Organization (WHO) Classification (12), consisting mainly or entirely of small round cells with scant cytoplasm. Morphological and immunohistochemical diagnosis of SCCO is frequently challenging due to the large number of tumors that must be differentially diagnosed, such as sex cord-stromal tumors and neoplasms in the category of small round blue cell tumors (13). Although some clinical guidelines have previously been published, the clinical management of SCCO has varied considerably due to the lack of a recognized first-line treatment strategy. Its prognosis remains poor, with a long-term survival rate of 10-20% based on a study of 150 cases conducted in 1994 (14). Most current studies are limited to case reports, with only two statistical studies based on large samples reported till date. The study, which enrolled 150 cases in 1994, investigated the effects of the Federation International of Gynecology and Obstetrics (FIGO) stage and treatment on the prognosis. Since the cases were diagnosed about 30 years ago, many crucial factors have changed over such a long time. Another study, which enrolled 180 cases, solely compared the overall survival between SCCO and small cell lung cancer (SCLC) based on the different stages, which did not make much sense for clinical guidance (15). Hence, the clinical characteristics associated with a poorer outcome of SCCO must be identified. As HGSOC is the most prevalent subtype of ovarian cancer, comparing its characteristics and prognosis with SCCO may have guiding significance for clinical practice. By far, there has been no clinical research comparing SCCO and HGSOC.

The Surveillance, Epidemiology, and End Results (SEER) database, established in the United States, is an official cancer database with population-based clinical survival data from registries covering 34.6% of the national population (16, 17). This study aimed to compare the clinical characteristics, treatment methods, and prognosis of SCCO and HGSOC in a large sample. Furthermore, independent risk factors for a poorer outcome of SCCO or both SCCO and HGSOC were identified.

## 2 Materials and methods

### 2.1 Data source

**Figure 1:**
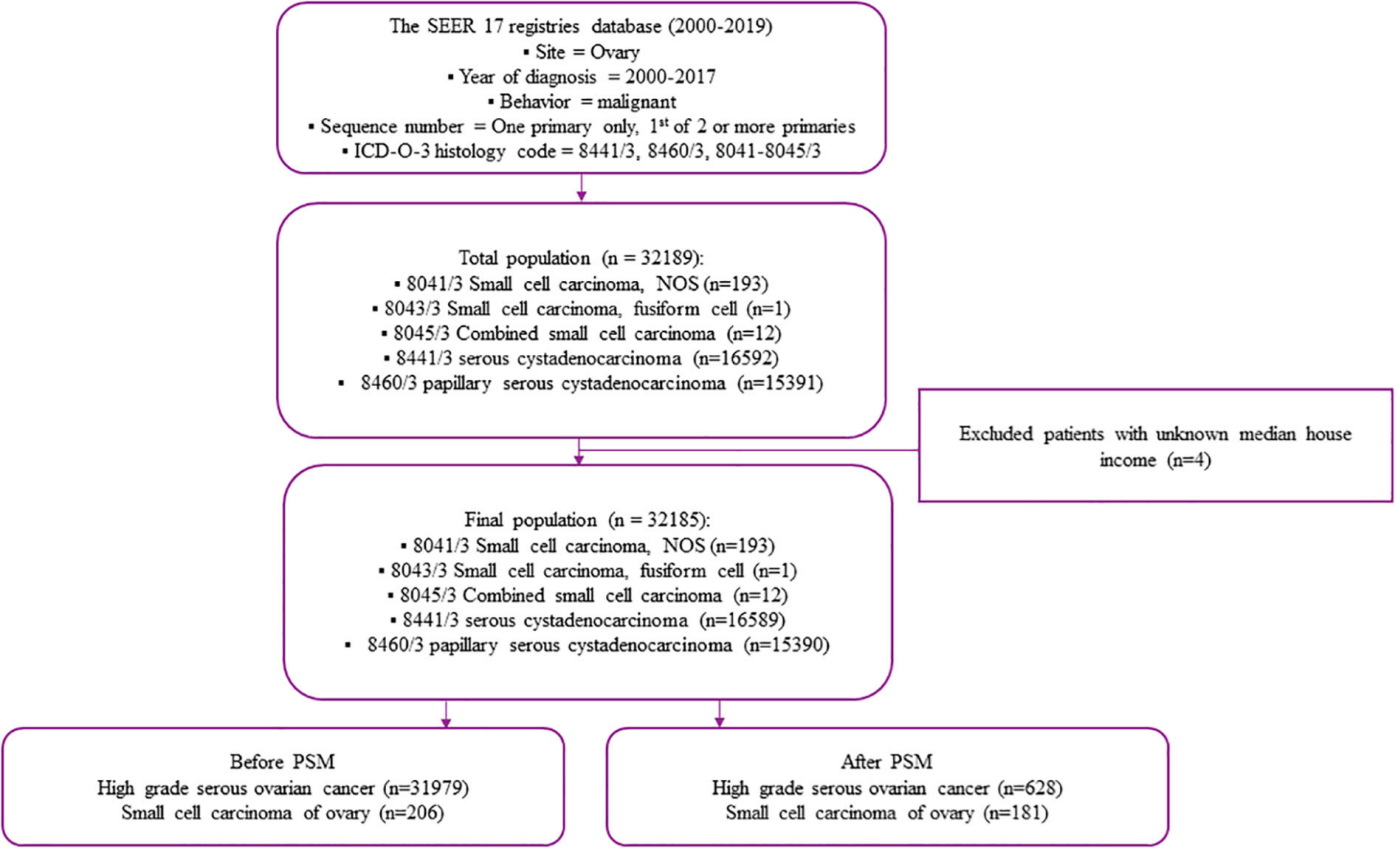
Study flow chart.

From 1998 to 2016, ovarian cancer in the SEER program was identified *via* the site-specific International Classification of Oncological Diseases 3 (ICD-O-3) code C56.9. The diagnosis of SCCO was determined using the ICD-O-3 codes 8041-8045/3, while the diagnosis of HGSOC was determined using the ICD-O-3 codes 8460/3 (Papillary serous cystadenocarcinoma) and 8441/3(Serous cystadenocarcinoma, NOS). The number of primary tumors was identified using the sequence number for a single primary or the first of two or more primaries. Exclusion criteria were (1): the tumor was not primary; (2) the case was without complete follow-up data. Finally, 31,979 patients with HGSOC and 206 patients with SCCO were selected. The codes for case collection were complied with the guidelines of the SEER database coding and staging manual (**Figure 1**).

### 2.2 Clinical information

Variables abstracted from the SEER database included demographic information (e.g., patient ID, age at diagnosis, median household income, and marital status), tumor characteristics (e.g., tumor size, laterality, grade, FIGO stage, SEER stage, sequence number, serum carbohydrate antigen 125 [CA125], and lymph node status), treatment (e.g., surgery status, radiotherapy, and chemotherapy), and follow-up for survival (survival months, cause-specific death, and vital status). Among them, SEER stage refers to Summary Stage which is derived from Collaborative Stage (CS) for 2004+ and Extent of Disease (EOD) from 1998-2003. It is a simplified version of stage: in situ, localized, regional, distant, & unknown. For more information, see https://seer.cancer.gov/seerstat/variables/seer/lrd-stage/.

Demographic information was recorded as race (white, black, other, and unknown) and marital status (single/unmarried, married, divorced/separated/widowed, and un-known). Tumor characteristics were recorded as FIGO stage (I, II, III, IV, or unknown), the number of primary tumors (one primary only versus 1st of 2 or more primaries), Grade (I/II, III/IV, or unknown), lymph node status (negative, positive, or unknown), and CA125 status (negative, positive, or unknown). Treatment was as follows: surgery (yes vs. no), chemotherapy (yes vs. no/unknown), and radiation therapy status (yes vs. no).

### 2.3 Statistical analyses

**Figure 2:**
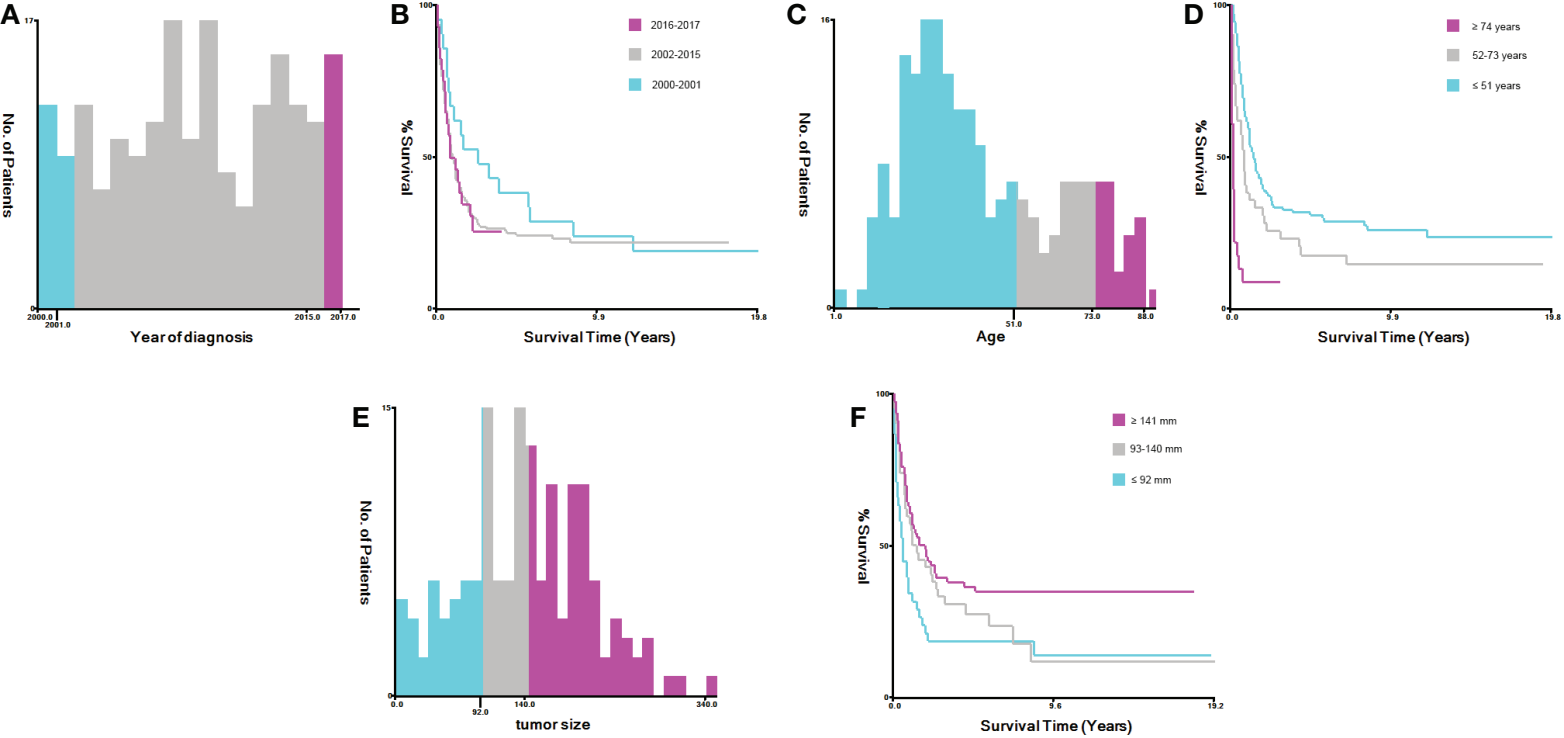
Identification of optimal cut-off values for year of diagnosis, age and tumor size *via* X-tile software analysis. **(A, B)** Optimal minimum and maximum cut-off value 2001 and 2005 for year of diagnosis and its survival curves. **(C, D)** Optimal minimum and maximum cut-off values 57 and 73 years for age and its survival curves. **(E, F)** Optimal minimum and maximum cut-off value 59 and 176 mm for tumor size and its survival curves.

X-tile software version 3.6.1 was used to calculate the optimum cutoff value for converting continuous variables (e.g., age at diagnosis, year of diagnosis, and tumor size) into categorical variables. The variable age at diagnosis was then categorized into three groups: ≤ 51, 52-73, and ≥ 74 years. The variable year of diagnosis was grouped as 2000-2001, 2002-2015, and 2016-2017, while tumor size was grouped into ≤ 92, 93-140, and ≥ 141 mm groups (**Figure 2**). As the clinical characteristics were heterogeneous between cases with SCCO and HGSOC in the SEER database *via* the analysis of Chi-square or Fisher’s exact tests, the propensity score matching (PSM) was used to adjust the base-line characteristics of patients with SCCO and HGSOC. Using the R package ‘‘MatchIt’’ version 4.1.0, the following PSM settings were performed: 1-to-5 pairing, nearest neighbor methods, and a caliper of 0.05 (18). The propensity score model included all the aforementioned variables. Overall survival (OS) was defined as the time interval between diagnosis to death from any cause. Cancer-specific survival (CSS) was defined as the duration between diagnosis and death caused by ovarian cancer. These two indexes were the outcome endpoints of the present study. The survival plot was constructed *via* Kaplan-Meier analysis, and the comparison between patients with SCCO and HGSOC before and after PSM was made *via* log-rank test. Univariate and multivariate Cox regression analyses were employed to determine the potential prognostic variables on the OS and CSS of patients with SCCO.

To determine whether different pathological types and clinical characteristics can combinedly predict the prognosis of patients with SCCO or HGSOC after PSM, least absolute shrinkage and selection operator (LASSO)-COX analyses were performed to determine the optimal weighting coefficients for these features and build a model. LASSO-COX regression models for OS and CSS were built by performing ten-fold cross-validation using the “glmnet” package in R software. Moreover, the optimal value of the lambda parameter was 0.026 and 0.038 in OS and CSS, respectively.

Based on the above model, ROC analysis of the follow-up outcomes and risk scores over ten years was performed using the “pROC” package in R and evaluated the area under the curve (AUC) and confidence interval (CI). Based on the optimal cutoff or median of risk scores, patients were categorized into high- and low-risk groups, and the prognostic differences between the two groups were further analyzed using the ‘survival’ package. The significance of the difference in prognosis between the two groups was assessed using the log-rank test.

Finally, survival data from the LASSO-COX analysis was integrated through the R ‘rms’ package to construct nomograms and predict the OS and CSS of patients at 1, 3, and 5 years. By integrating multiple predictors and plotting multiple lines to scale, a nomogram easily calculates the risk of disease or an individual’s probability of survival and uses the C-index to assess the power of the nomogram.

## 3 Results

### 3.1 Comparison of the baseline clinical characteristics between SCCO and HGSOC

A total of 41,458 patients, including 31,979 patients with HGSOC and 206 with SCCO in the SEER database, were enrolled in the study (**Figure 1**). The differences in most of the baseline clinical characteristics between HGSOC and CENO were statistically significant (p <0.05) (**Table 1**). The year of diagnosis, surgery/chemotherapy status, and race of the patients with HGSOC were similar to those with SCCO. In terms of laterality, the SCCO group had a lower percentage of bilateral (13.1% vs. 51.0%) and a higher percent-age of unilateral (78.2% vs. 39.5%) than the HGSOC group (p <0.001). Compared to the patients with HGSOC, patients with SCCO were more likely to be aged ≤ 51 years (68.4% vs. 20.4%, p <0.001), single (43.7% vs. 15.6%, p <0.001), and have a median house income less than $70,000 (69.4% vs. 57.0%, p <0.001). Patients with SCCO were more likely to receive radiotherapy (6.8% vs. 0.8%, p <0.001), have negative CA125 (8.7% vs. 4.0%, p <0.001) and tumors ≥ 141mm (38.3% vs. 9.7%, p <0.001). Furthermore, patients with SCCO were less likely to have stage III-IV disease (61.7% vs. 77.0%, p <0.001) and distant tumors in SEER stage (59.2% vs. 77.6%, p <0.001). Moreover, the tumor grade distribution differed between patients with HGSOC and SCCO (p <0.001).

**Table 1:** Demographic and clinical characteristics comparing small cell carcinoma of the ovary and high-grade serous ovarian cancer.

Subject Characteristic	Before propensity score matching		P-value	After propensity score matching		SD
	HGSOC	SCCO		HGSOC	SCCO	
	N(%)	N(%)		N(%)	N(%)	
All	31979	206		628	181	
Year of diagnosis						
2000-2001	3117 (9.7)	21 (10.2)	0.851	66 (10.5)	18 (9.9)	0.052
2002-2015	24880 (77.8)	157 (76.2)		486 (77.4)	138 (76.2)	
2016-2017	3982 (12.5)	28 (13.6)		76 (12.1)	25 (13.8)	
Laterality						
Bilateral	16313 (51.0)	27 (13.1)	<0.001	92 (14.6)	27 (14.9)	0.06
Left	6003 (18.8)	73 (35.4)		221 (35.2)	61 (33.7)	
Only one side - side unspecified	290 (0.9)	3 (1.5)		8 (1.3)	3 (1.7)	
Paired site, but no information concerning laterality	3038 (9.5)	18 (8.7)		69 (11.0)	18 (9.9)	
Right	6335 (19.8)	85 (41.3)		238 (37.9)	72 (39.8)	
Surgery status						
No surgery	3492 (10.9)	43 (20.9)	<0.001	136 (21.7)	41 (22.7)	0.024
Surgery	28447 (89.0)	163 (79.1)		492 (78.3)	140 (77.3)	
Unknown	40 (0.1)	0 (0.0)		0 (0.0)	0 (0.0)	
Radiation therapy						
No radiation	31709 (99.2)	192 (93.2)	<0.001	607 (96.7)	177 (97.8)	0.069
Radiation	270 (0.8)	14 (6.8)		21 (3.3)	4 (2.2)	
Chemotherapy status						
Chemotherapy	6676 (20.9)	50 (24.3)	0.267	168 (26.8)	48 (26.5)	0.005
No chemotherapy/Unknown	25303 (79.1)	156 (75.7)		460 (73.2)	133 (73.5)	
Number of primary tumors						
1st of 2 or more primaries	2453 (7.7)	12 (5.8)	0.389	42 (6.7)	12 (6.6)	0.002
One primary only	29526 (92.3)	194 (94.2)		586 (93.3)	169 (93.4)	
Race						
Black	2182 (6.8)	16 (7.8)	0.225	38 (6.1)	16 (8.8)	0.116
Other (American Indian/AK Native, Asian/Pacific Islander)	2526 (7.9)	18 (8.7)		54 (8.6)	17 (9.4)	
Unknown	85 (0.3)	2 (1.0)		5 (0.8)	1 (0.6)	
White	27186 (85.0)	170 (82.5)		531 (84.6)	147 (81.2)	
Marital status at diagnosis						
DSW	8794 (27.5)	30 (14.6)	<0.001	113 (18.0)	28 (15.5)	0.089
Married	16988 (53.1)	79 (38.3)		264 (42.0)	74 (40.9)	
Single	4997 (15.6)	90 (43.7)		226 (36.0)	72 (39.8)	
Unknown	1200 (3.8)	7 (3.4)		25 (4.0)	7 (3.9)	
Median household income ($)						
<50,000	3542 (11.1)	35 (17.0)	<0.001	106 (16.9)	29 (16.0)	0.036
>=70,000	13766 (43.0)	63 (30.6)		207 (33.0)	58 (32.0)	
50,000-69,999	14671 (45.9)	108 (52.4)		315 (50.2)	94 (51.9)	
Age (years)						
<=51	6532 (20.4)	141 (68.4)	<0.001	354 (56.4)	116 (64.1)	0.165
>=74	6844 (21.4)	23 (11.2)		107 (17.0)	23 (12.7)	
52-73	18603 (58.2)	42 (20.4)		167 (26.6)	42 (23.2)	
Grade						
I/II	4524 (14.1)	1 (0.5)	<0.001	8 (1.3)	1 (0.6)	0.083
III/IV	20364 (63.7)	107 (51.9)		327 (52.1)	92 (50.8)	
Unknown	7091 (22.2)	98 (47.6)		293 (46.7)	88 (48.6)	
Seer stage						
Distant	24812 (77.6)	122 (59.2)	<0.001	406 (64.6)	114 (63.0)	0.051
Localized	1706 (5.3)	26 (12.6)		68 (10.8)	19 (10.5)	
Regional	4933 (15.4)	53 (25.7)		140 (22.3)	43 (23.8)	
Unknown	528 (1.7)	5 (2.4)		14 (2.2)	5 (2.8)	
Stage						
I	2771 (8.7)	44 (21.4)	<0.001	105 (16.7)	31 (17.1)	0.086
II	2269 (7.1)	15 (7.3)		51 (8.1)	14 (7.7)	
III	15489 (48.4)	65 (31.6)		224 (35.7)	58 (32.0)	
IV	9140 (28.6)	62 (30.1)		188 (29.9)	59 (32.6)	
Unknown	2310 (7.2)	20 (9.7)		60 (9.6)	19 (10.5)	
tumor size (mm)						
<=92	11859 (37.1)	38 (18.4)	<0.001	136 (21.7)	37 (20.4)	0.1
>=141	3108 (9.7)	79 (38.3)		176 (28.0)	59 (32.6)	
93-140	5081 (15.9)	42 (20.4)		141 (22.5)	38 (21.0)	
Unknown	11931 (37.3)	47 (22.8)		175 (27.9)	47 (26.0)	
Lymph nodes status						
negative	7586 (23.7)	59 (28.6)	0.18	177 (28.2)	46 (25.4)	0.063
positive	7094 (22.2)	38 (18.4)		113 (18.0)	34 (18.8)	
Unknown	17299 (54.1)	109 (52.9)		338 (53.8)	101 (55.8)	
CA125						
negative	1283 (4.0)	18 (8.7)	<0.001	41 (6.5)	16 (8.8)	0.095
positive	19475 (60.9)	92 (44.7)		291 (46.3)	85 (47.0)	
Unknown	11221 (35.1)	96 (46.6)		296 (47.1)	80 (44.2)	

### 3.2 Propensity score matching and survival analysis

The propensity score matching (PSM) method was used to balance the baseline clinical characteristics between patients with SCCO and HGSOC (all standard deviations ≤ 0.05; **Table 1**). Ultimately, 809 patients after PSM (SCCO group, n = 628; HGSOC group, n = 181) were included in the following analysis.

**Figure 3:**
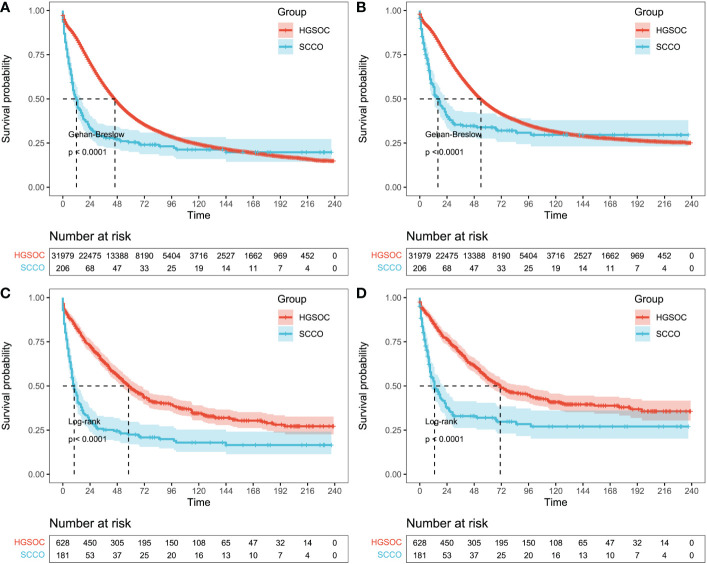
Survival outcomes before and after propensity-score matching. **(A)** Overall survival and **(B)** cancer-specific survival based on patients with HGSOC or SCCO before propensity-score matching. Gehan-Breslow tests were used to generate P-values. **(C)** Overall survival and **(D)** cancer-specific survival based on patients with HGSOC or SCCO after propensity-score matching. Log-rank tests were used to generate P-values. HGSOC, high grade serous ovarian cancer; SCCO, Small cell carcinoma of the ovary.

Patients with SCCO and HGSOC before and after PSM were assessed for Kaplan-Meier survival analysis. A total of 32,185 patients (SCCO group, n = 206; HGSOC group, n = 31,979) were enrolled in the analysis before PSM. The median OS was 12.0 months in the SCCO group and 45.0 months in the HGSOC group, while the median CSS was 16.0 months in the SCCO group and 53.0 months in the HGSOC group. A poorer outcome was observed in the SCCO group (1-year OS rates: 47.5% vs. 84.1%, 3-year OS rates: 29.1% vs. 58.6%, and 5-year OS rates: 26.7% vs. 43.9%; 1-year CSS rates: 59.2% vs. 87.1%, 3-year CSS rates: 41.8% vs. 65.0%, and 5-year CSS rates: 40.8% vs. 52.4%; p <0.001) than in the HGSOC group (**Figures 3A, B**). A similar conclusion could be obtained for patients with SCCO and HGSOC after PSM. The median OS was 9.0 months in the SCCO group and 58.0 months in the HGSOC group, while the median CSS was 13.0 months in the SCCO group and 70.0 months in the HGSOC group. OS and CSS rate were worse in patients with SCCO than in those with HGSOC (1-year OS rates: 44.8% vs. 82.6%, 3-year OS rates: 26.0% vs. 64.5%, and 5-year OS rates: 23.8% vs. 51.4%; 1-year CSS rates: 55.8% vs. 85.7%, 3-year CSS rates: 40.3% vs. 70.4%, and 5-year CSS rates: 39.8% vs. 58.9%, p <0.001) (**Figures 3C, D**).

### 3.3 Univariate and multivariate analysis

Univariate and multivariate Cox regression analyses were performed to determine the potential clinical characteristics which may influence the prognosis of patients with SCCO (n = 206).

In univariate regression analysis, age, marital status at diagnosis, laterality, surgery status, chemotherapy/radiotherapy, FIGO stage, SEER stage, lymph node status, and tumor size were prognostic risk factors for both OS and CSS in patients with SCCO. The number of primary tumors remained a prognostic factor for CSS, while median household income remained a prognostic factor for OS (p <0.05, **Table 2**).

**Table 2:** Univariable Cox Regression for analyzing the associated factors for small cell carcinoma of the ovary.

Subject characteristics	Overall survival (OS)		Cancer-specific survival (CSS)	
	HR (95%CI)	P-value	HR (95%CI)	P-value
Year of diagnosis				
2000-2001	Reference		Reference	
2002-2015	1.28(0.77~2.13)	0.347	1.96(0.99~3.89)	0.053
2016-2017	1.33(0.69~2.56)	0.389	2.19(0.98~4.88)	0.057
Laterality				
Bilateral	Reference		Reference	
Left	0.5(0.31~0.8)	**0.004**	0.55(0.33~0.94)	**0.028**
Only one side - side unspecified	8.96(2.53~31.65)	**0.001**	12.27(3.34~45.08)	**<0.001**
Paired site, but no information concerning laterality	1.84(1~3.38)	**0.05**	0.88(0.37~2.1)	0.774
Right	0.43(0.27~0.68)	**<0.001**	0.52(0.31~0.88)	**0.015**
Surgery status				
No surgery	Reference		Reference	
Surgery	0.26(0.18~0.38)	**<0.001**	0.35(0.22~0.54)	**<0.001**
Radiation therapy				
No radiation	Reference		Reference	
Radiation	0.35(0.15~0.79)	**0.012**	0.36(0.15~0.89)	**0.027**
Chemotherapy status				
Chemotherapy	Reference		Reference	
No chemotherapy/Unknown	0.43(0.3~0.61)	**<0.001**	0.49(0.33~0.74)	**0.001**
Number of primary tumors				
1st of 2 or more primaries	Reference		Reference	
One primary only	2(0.93~4.28)	0.076	3.65(1.16~11.52)	**0.027**
Race				
Black	Reference		Reference	
Other (American Indian/AK Native, Asian/Pacific Islander)	0.76(0.35~1.64)	0.487	0.89(0.37~2.14)	0.799
Unknown	NA	0.994	NA	NA
White	0.92(0.53~1.6)	0.772	1.05(0.55~2.02)	0.873
Marital status at diagnosis				
DSW	Reference		Reference	
Married	0.71(0.45~1.12)	0.144	0.75(0.45~1.28)	0.295
Single	0.54(0.34~0.85)	**0.008**	0.56(0.33~0.96)	**0.034**
Unknown	0.67(0.26~1.76)	0.422	0.9(0.34~2.4)	0.83
Median household income ($)				
<50,000	Reference		Reference	
>=70,000	0.7(0.45~1.1)	0.118	0.64(0.38~1.06)	0.084
50,000-69,999	0.62(0.41~0.93)	**0.022**	0.65(0.41~1.03)	0.064
Age (years)				
<=51	Reference		Reference	
>=74	5.36(3.3~8.69)	**<0.001**	4.25(2.42~7.46)	**<0.001**
52-73	1.55(1.05~2.28)	**0.026**	1.29(0.83~2.02)	0.261
Grade				
I/II	Reference		Reference	
III/IV	0.25(0.03~1.79)	0.166	NA	NA
Unknown	0.31(0.04~2.22)	0.242	NA	NA
Seer stage				
Distant	Reference		Reference	
Localized	0.17(0.08~0.33)	**<0.001**	0.2(0.1~0.42)	**<0.001**
Regional	0.42(0.29~0.62)	**<0.001**	0.52(0.34~0.78)	**0.002**
Unknown	0.82(0.3~2.22)	0.695	0.78(0.25~2.47)	0.671
Stage				
I	Reference		Reference	
II	2.81(1.36~5.81)	**0.005**	2.44(1.13~5.3)	**0.024**
III	3.85(2.28~6.5)	**<0.001**	3.34(1.93~5.77)	**<0.001**
IV	5.17(3.05~8.76)	**<0.001**	3.68(2.08~6.5)	**<0.001**
Unknown	3.39(1.72~6.69)	**<0.001**	3.04(1.48~6.21)	**0.002**
tumor size (mm)				
<=92	Reference		Reference	
>=141	0.48(0.31~0.75)	**0.001**	0.48(0.3~0.79)	**0.003**
93-140	0.66(0.4~1.07)	0.09	0.68(0.4~1.14)	0.145
Unknown	0.98(0.62~1.56)	0.939	0.77(0.45~1.31)	0.334
Lymph nodes status				
negative	Reference		Reference	
positive	1.88(1.13~3.14)	**0.015**	1.67(0.96~2.89)	0.07
Unknown	3.01(2.02~4.49)	**<0.001**	2.47(1.61~3.8)	**<0.001**
CA125				
negative	Reference		Reference	
positive	1.55(0.84~2.86)	0.16	1.72(0.85~3.48)	0.128
Unknown	1.33(0.72~2.45)	0.359	1.41(0.7~2.84)	0.34

The values under 0.05 were represented in bold.

In multivariate regression analysis, unilateral (only one side/right side vs. bilateral, OR 6.34/0.59, p = 0.009/0.048), surgery status (surgery vs. no surgery, OR 0.54, p = 0.026), radiation therapy (radiation vs. no radiation, OR 0.54, p = 0.026), chemotherapy (no chemotherapy/unknown vs. chemotherapy, OR 0.35, p <0.001), older age (≥ 74 vs. ≤ 57, OR 2.55, p = 0.004), and stage II disease (OR 2.49 compared to stage I disease, p = 0.048) were determined as independent characteristics associated with OS of SCCO. Meanwhile unilateral (only one side vs. bilateral, OR 14.35, p <0.001), surgery status (surgery vs. no surgery, OR 0.40, p = 0.004), radiation therapy (radiation vs. no radiation, OR 0.29, p = 0.012), chemotherapy (no chemotherapy/unknown vs. chemotherapy, OR 0.44, p = 0.001), number of primary tumors (one primary only vs. first of 2 or more primaries, OR 0.003, p = 0.003), older age (58-73 vs. ≤ 57, OR 0.38, p = 0.002), stage II disease (OR 2.67 compared to stage I disease, p = 0.040), and larger tumor size (≥ 141 mm vs. ≤ 92 mm, OR 0.46, p = 0.012) were determined as independent characteristics associated with CSS of SCCO.

### 3.4 Construction of predictive models for OS and CSS using prognostic factors

**Figure 4:**
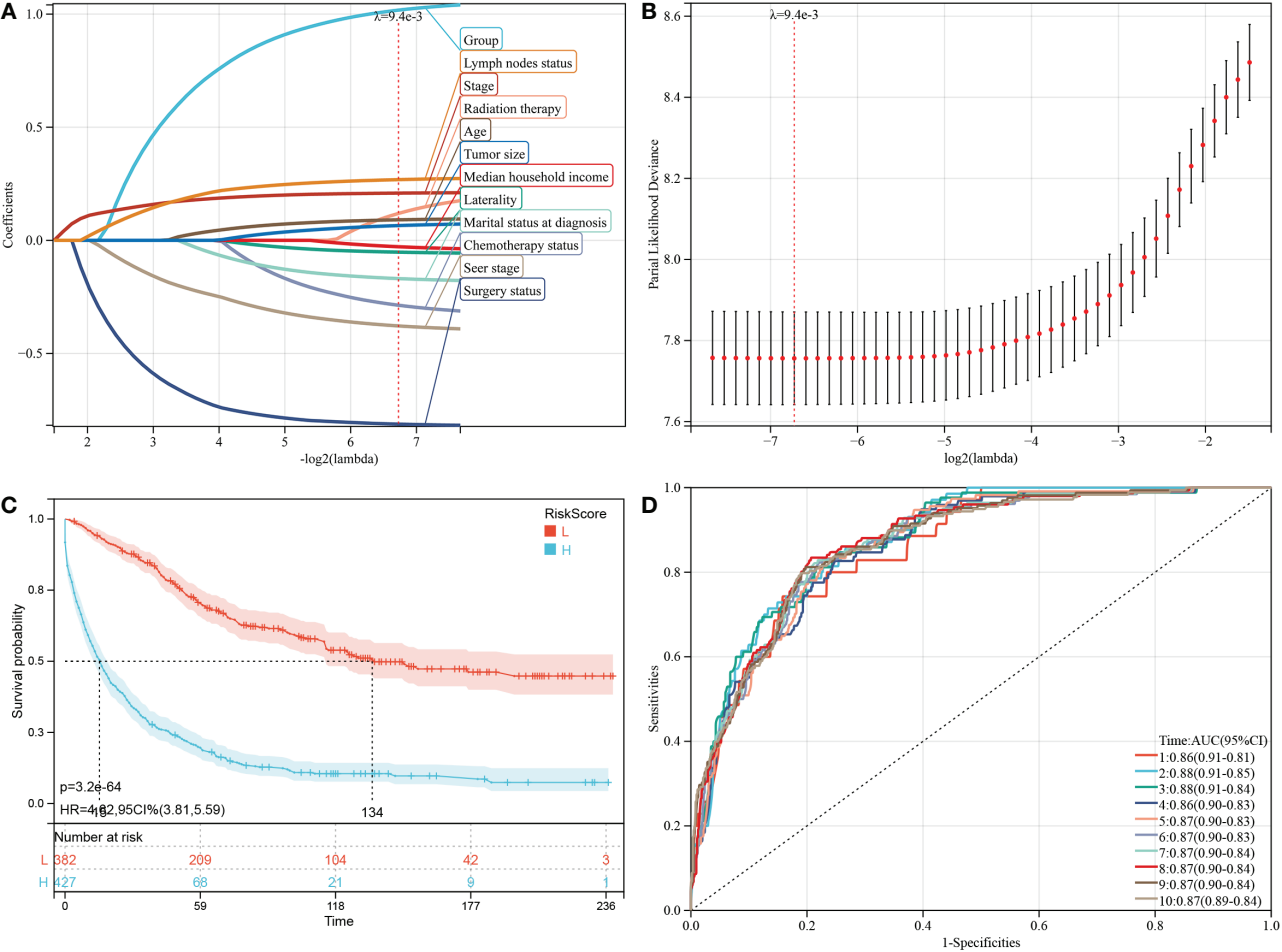
Construction and evaluation of OS associated predictive models. **(A, B)** The LASSO coefficient profiles and LASSO deviance profiles, respectively, show the optimal λ value and risk factors. **(C)** KM survival curves of OS according to the risk score perform that prognosis of low-risk group is significantly better than high-risk score group. **(D)** ROC curves of OS at 1-10 years according to the risk score in the predictive model data sets.

**Figure 5:**
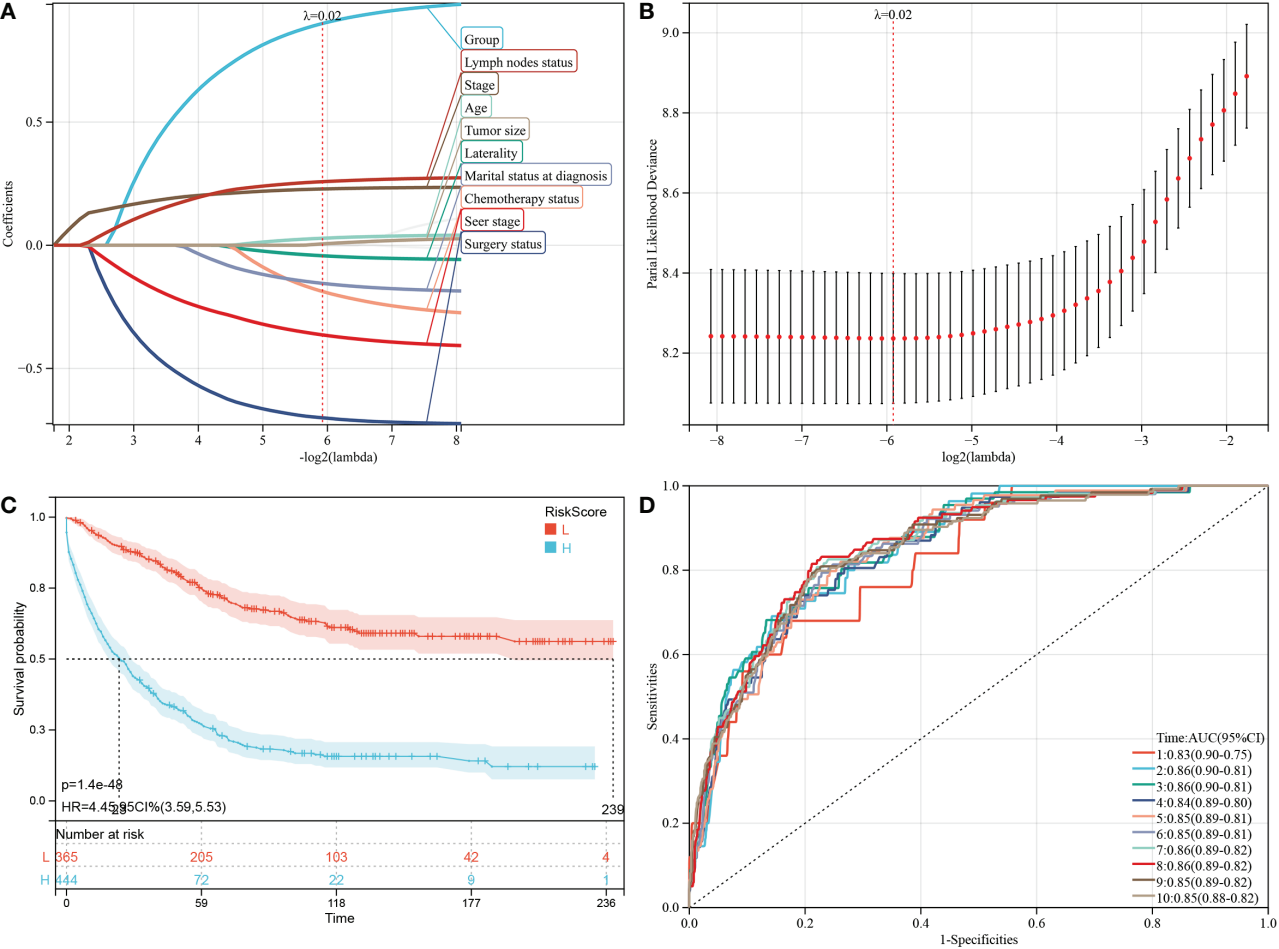
Construction and evaluation of CSS associated predictive models. **(A, B)** are the LASSO coefficient profiles and LASSO deviance profiles, respectively, which show the optimal λ value and risk factors. **(C)** KM survival curves of CSS according to the risk score perform that prognosis of low-risk group is significantly better than high-risk score group. **(D)** ROC curves of CSS at 1-10 years according to the risk score in the predictive model data sets.

Based on the analysis of the above-mentioned prognostic factors, it is essential to investigate whether the HGSOC and SCCO groups impact the prognosis of patients. Therefore, LASSO-COX analysis was performed after univariate COX analysis to construct predictive OS and CSS models. The LASSO-COX regression models of OS (**Figures 4A, B**) or CSS (**Figures 5A, B**) were constructed, integrating the significant prognostic factors (**Table 3**) and group information, respectively. After 10-fold cross-validation, the optimal λ values 9.4e-3 and 2e-2 were obtained in OS and CSS models, respectively. Finally, the group and 11 prognostic factors were determined in the predictive model of OS, including laterality, surgery status, radiation therapy, chemotherapy status, marital status at diagnosis, median household income, age, seer stage, FIGO stage, tumor size, and lymph nodes status (**Figures 4A, B**). In the predictive model of OS, the risk score of OS was generated using the following formula:

**Table 3:** Multivariable Cox Regression for analyzing the associated factors for small cell carcinoma of the ovary.

Subject characteristics	Overall survival (OS)		Cancer-specific survival (CSS)	
	HR (95%CI)	P-value	HR (95%CI)	P-value
Laterality				
Bilateral	Reference		Reference	
Left	0.68(0.4~1.14)	0.142	0.74(0.41~1.31)	0.301
Only one side - side unspecified	6.34(1.58~25.46)	**0.009**	14.35(3.41~60.38)	**<0.001**
Paired site, but no information concerning laterality	0.96(0.45~2.05)	0.914	0.42(0.16~1.14)	0.09
Right	0.59(0.35~0.99)	**0.048**	0.66(0.37~1.17)	0.154
Surgery status				
No surgery	Reference		Reference	
Surgery	0.54(0.31~0.93)	**0.026**	0.4(0.22~0.75)	**0.004**
Radiation therapy				
No radiation	Reference		Reference	
Radiation	0.42(0.17~1)	**0.049**	0.29(0.11~0.76)	**0.012**
Chemotherapy status				
Chemotherapy	Reference		Reference	
No chemotherapy/Unknown	0.35(0.23~0.53)	**<0.001**	0.44(0.27~0.72)	**0.001**
Number of primary tumors				
1st of 2 or more primaries			Reference	
One primary only			6.4(1.9~21.6)	**0.003**
Marital status at diagnosis				
DSW	Reference		Reference	
Married	1.52(0.79~2.93)	0.208	1.41(0.72~2.77)	0.312
Single	1.19(0.61~2.34)	0.607	1.04(0.52~2.08)	0.91
Unknown	1.7(0.56~5.16)	0.352	1.85(0.61~5.62)	0.278
Median household income ($)				
<50,000	Reference			
>=70,000	0.78(0.47~1.3)	0.34		
50,000-69,999	0.64(0.41~1)	0.052		
Age (years)				
<=51	Reference		Reference	
>=74	2.55(1.21~5.38)	**0.014**	1.93(0.85~4.36)	0.114
52-73	0.63(0.37~1.08)	0.096	0.38(0.2~0.71)	**0.002**
Seer stage				
Distant	Reference		Reference	
Localized	0.38(0.14~1.05)	0.063	0.36(0.12~1.05)	0.062
Regional	0.55(0.28~1.1)	0.089	0.52(0.25~1.07)	0.077
Unknown	1.15(0.34~3.94)	0.824	0.99(0.24~4.06)	0.994
Stage				
I	Reference		Reference	
II	2.49(1.01~6.13)	**0.048**	2.67(1.05~6.8)	**0.04**
III	2.22(0.92~5.35)	0.076	2.09(0.85~5.19)	0.11
IV	1.53(0.56~4.16)	0.405	1.18(0.41~3.38)	0.762
Unknown	1.01(0.4~2.53)	0.99	1.11(0.42~2.91)	0.836
tumor size (mm)				
<=92	Reference		Reference	
>=141	0.77(0.45~1.32)	0.345	0.46(0.25~0.84)	**0.012**
93-140	0.77(0.43~1.39)	0.387	0.56(0.3~1.07)	0.079
Unknown	0.91(0.52~1.57)	0.727	0.59(0.3~1.16)	0.125
Lymph nodes status				
negative	Reference		Reference	
positive	0.94(0.5~1.76)	0.854	0.69(0.36~1.33)	0.274
Unknown	1.38(0.83~2.29)	0.215	1.17(0.68~2)	0.573

The values under 0.05 were represented in bold.

Risk score = 1.018 × Group-0.052 × Laterality-0.813 × Surgery status + 0.119 × Radiation therapy-0.287 × Chemotherapy status-0.169 × Marital status at diagnosis-0.028 × Median household income + 0.091 × Age-0.378 × Seer stage + 0.209 × Stage + 0.065 × Tu-mor size + 0.268 × Lymph nodes status.

Furthermore, 809 patients screened by PSM were subjected to survival analysis according to the risk score. The optimal cutoff value was determined by -0.343 for OS model. The included patients could be classified into high-risk and low-risk groups based on their cutoff values. The Kaplan-Meier curve analysis revealed that the predictive model of OS could distinguish patients with good or bad prognoses. The high-risk group manifested a shorter OS than the low-risk group (p = 3.2e-64) (**Figure 4C**). Time-dependent ROC analysis showed that AUC of risk score for the prediction of 1-10 year OS was 0.86, 0.88, 0.88 0.86, 0.87, 0.87, 0.87, 0.87, 0.87, and 0.87, respectively (**Figure 4D**).

Subsequently, the factors in the CSS predictive model included group, laterality, surgery status, chemotherapy status, marital status at diagnosis, age, seer stage, FIGO stage, tumor size, and lymph nodes status (**Figures 5A, B**), and the risk score of CSS was generated using the following formula:

Risk score = 0.900 × Group-0.043 × Laterality-0.700 × Surgery status-0.188 × Chemotherapy status-0.155 × Marital status at diagnosis + 0.028 × Age-0.364 × Seer stage + 0.228 × Stage + 0.006 × Tumor size + 0.258 × Lymph nodes status.

Similarly, the high-risk group manifested a shorter CSS than the CSS of the low-risk group in **Figure 5C** (p = 1.4e-48). In predictive model of CSS, the AUC value of the risk score for predicting 1-10 year CSS were 0.83, 0.86, 0.86, 0.84, 0.85, 0.85, 0.86, 0.86, 0.85, and 0.85, respectively (**Figure 5D**). 

**Figure 6:**
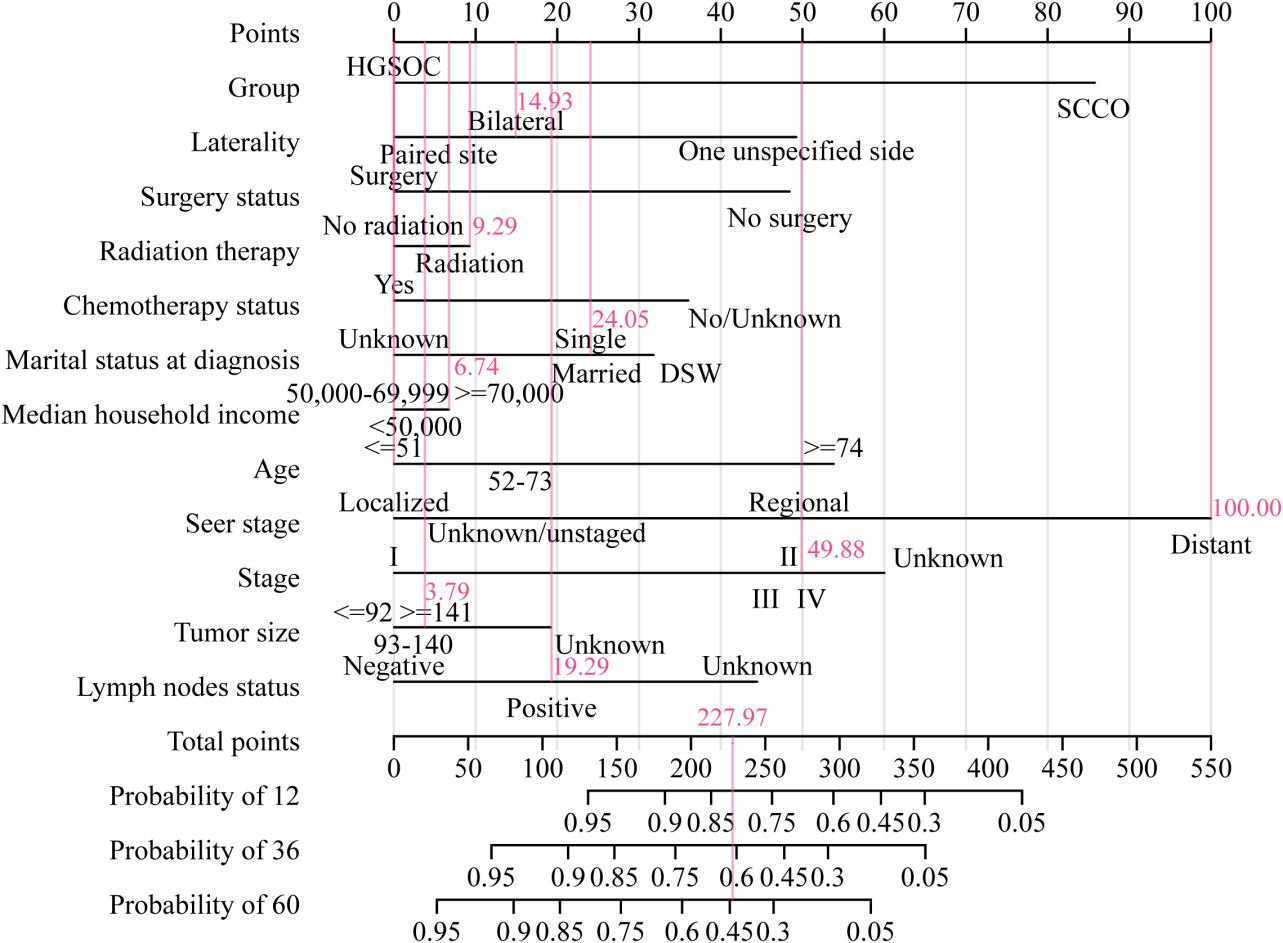
The nomogram of OS associated predictive models. The sum of the scores represented by the pink arrows represents the survival probability corresponding to 1, 3, and 5 years.

**Figure 7:**
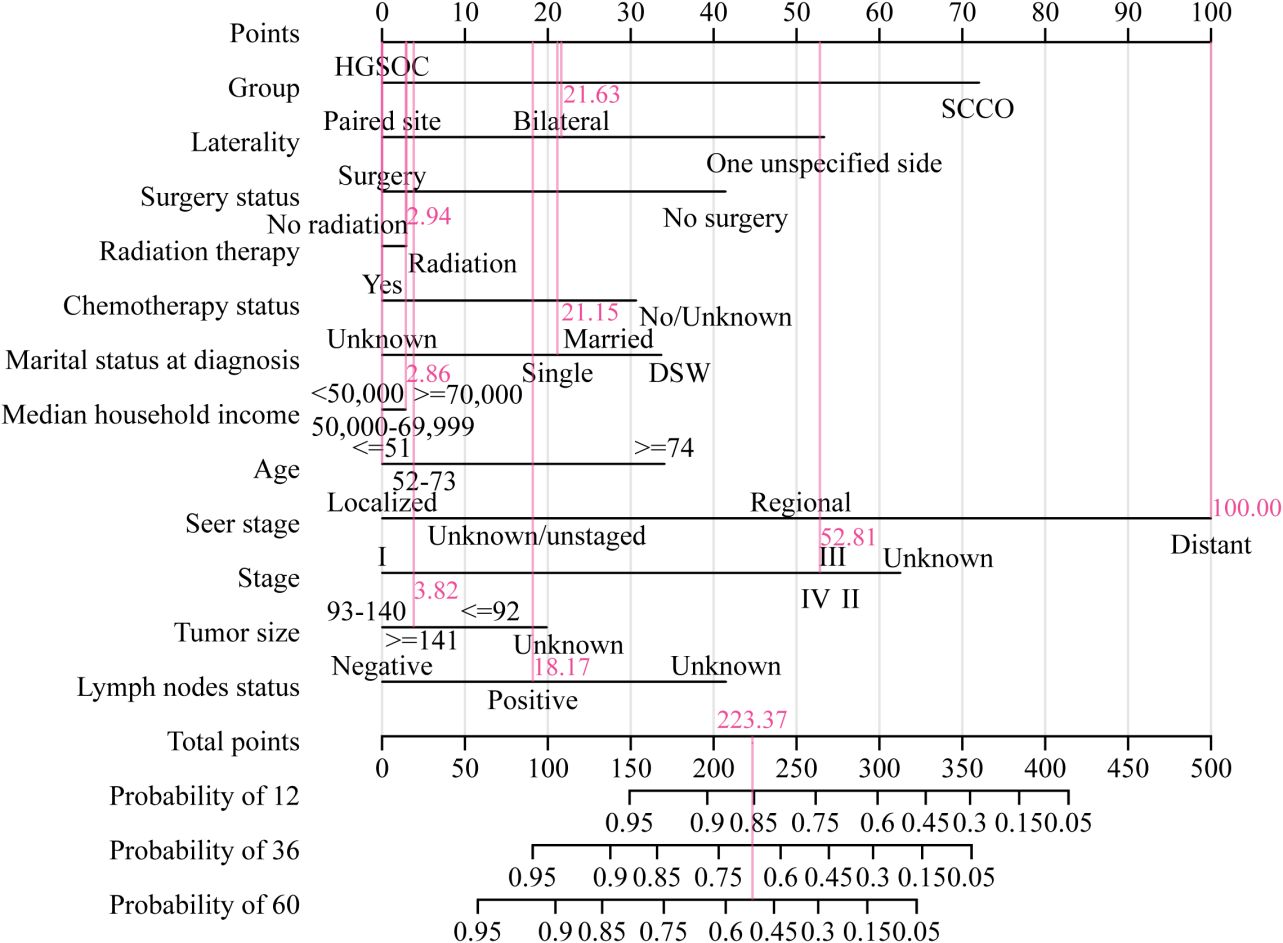
The nomogram of CSS associated predictive models. The sum of the scores represented by the pink arrows represents the survival probability corresponding to 1, 3, and 5 years.

The nomogram and calibration curves were used in our study to illustrate the predictive model of OS (**Figure 6**) or CSS (**Figure 7**) more vividly and improve the practicality of this model. The score of each characteristic was determined using the aforementioned scale. The sum of the scores of these characteristics was defined as the final score. The perpendicular line from the total point axis to the two-outcome axis allowed us to predict the prognosis of 1-, 3-, and 5-year OS or CSS for OC patients.

## 4 Discussion

SCCO is a rare and highly undifferentiated malignant tumor with an uncertain origin, accounting for about 0.01% of all ovarian tumors. According to the pathological features and immunohistochemical staining of tumors, SCCO was divided into SCCOHT and small cell carcinomas of the ovary-pulmonary type (SCCOPT) (5, 19, 20). Under the microscope, these subtypes were observed as small round densely arranged tumor cells; however, the clinical, histological, and imaging characteristics differed.

In the literature reports, SCCOHT was more prevalent in women under the age of 40, with an average age of 24 years. The age distribution of SCCOPT patients ranged from 28 to 85 years, and SCCOPT was more prevalent in perimenopausal and postmenopausal women, with a median age of 59 years (21). This finding was consistent with the conclusion drawn in the present study that SCCO patients younger than 51 years accounted for 64.1-68.4% of the total before or after propensity matching. Furthermore, the proportion of SCCO patients younger than 51 years (68.4%) was significantly higher than that of HGSOC patients (20.4%) before propensity matching. In the literature reports, SCCOHT often occurred in the unilateral ovary and was more common on the right side, which was round or irregular in shape. The typical MRI graphics of SCCOHT were dominated by solid components, with iso- and low-intensity on T1WI and high signal on T2WI, with obvious tumor heterogeneity and frequent hemorrhage and necrosis (14, 22). About 45% of SCCOPT showed unilateral ovarian involvement, with tumor size ranging from 4.5 to 26 cm (mean = 13.5 cm). The macroscopic observation of the tumor contains mucus; hence the lesions often appear as heterogeneous polycystic and solid structures on MRI (23), multiple separations can be seen between the cysts, and the solid components appear low signal on T1WI, slightly high signal on T2WI, and high signal on DWI (24–26). This study also suggested that unilateral SCCO accounted for 78.2% of total SCCO before PSM, with the right-sided tumor being the most prevalent. Furthermore, regardless of PSM, SCCO with tumor size greater than 141 mm had the largest proportion in this study group, and there was a significant difference between SCCO and HGSOC with tumor size greater than 141 mm before PSM. The initial epidemiological findings suggested that the data included in this study are consistent with previous cases.

In this study, significant differences in OS and CSS were found between HGSOC and SCCO in the SEER database through KM curve comparison. Moreover, after matching the epidemiological characteristics by the PSM method in HGSOC and SCCO, both OS and CSS of SCCO were found to be shorter than HGSOC, indicating that the prognosis of this case type was indeed worse than that of HGSOC. Therefore, this study further explored the factors affecting the prognosis of SCCO. Through univariate, multivariate, or LASSO-COX analysis, it was found that OS or CSS was associated with tumor laterality, whether surgery/radiotherapy/chemotherapy was received, the number of primary tumors, marital status, median household income, age, SEER stage, FIGO stage, tumor size, and lymph node metastasis. In this study, patients with unilateral tumors have been reported to have a significantly better prognosis than those with bilateral tumors (23, 27). At present, surgical treatment is the primary treatment method for most ovarian cancers, and the surgical procedures are mostly total hysterectomy with double adnexectomy, omentectomy, pelvic and para-aortic lymphadenectomy, and pelvic and abdominal implant foci debulking. Due to the high tumor malignancy and poor prognosis, there is a huge controversy over whether early-stage patients can preserve fertility (24, 28). Post-operative adjuvant chemotherapy with multiple drugs and radiotherapy is crucial to prolong survival. Estel et al. (21) retrospectively analyzed 47 SCCOHT patients and found that the patients whose primary adjuvant therapy included chemotherapy had a lower recurrence rate than those who did not receive chemotherapy, suggesting that chemotherapy is crucial in postoperative adjuvant therapy. However, there is currently no unified chemotherapy regimen for SCCO, and most chemotherapies are still based on cisplatin, supplemented by vincristine, etoposide, and other drugs. Recently, PD-1 inhibitors have also been used to treat patients with recurrent SCCO. After treatment, disease-free progression can last for 6-15 months, suggesting that PD-1/PD-L1 also has a certain effect on SCCO (29). However, there is no relevant data, which is also one of the criteria to be improved. Furthermore, it has been reported that early stage at diagnosis, age >30 years, normal serum calcium level, absence of large cell component in pathology, and tumor volume >10 cm (14, 21, 27) were all factors associated with better prognosis. Our findings confirmed that age >51 years and tumor size >141 mm are risk factors for SCCO. In addition, we first proposed that marital status and median house income were the independent prognostic factors for SCCO. In this study, the prognostic model constructed by these factors could significantly distinguish the prognosis of patients, and the nomogram could intuitively manifest the 1, 3, and 5-year survival probability of patients.

It is worth noting that the level of CA125 is not a risk or protective factor for SCCO in our study, although the literature reported that 75% of SCCOHT patients had elevated serum CA125 levels (21). Some cases reported that SCCOHT had familial inheritance (30), while CA125 could be one of the causes of SCCO diagnosis. However, our study found that CA125 did not determine prognosis, maybe because changes in CA125 are not linked to surgery/radiotherapy/chemotherapy status. In addition to CA125, 94% of SCCOHT patients showed immunohistochemical deletion of the SMARCA4 protein, resulting in mutations in the SMARCB1 gene and abnormal proliferation (31). These molecular abnormalities of SCCO have similar molecular and genetic manifestations to malignant rhabdoid tumors; hence some researchers believed that SCCOHT could be classified as ovarian malignant rhabdoid tumor and be diagnosed using SMARCA4 immunostaining and genetic testing as diagnostic criteria (22). However, the data on these indicators are lacking in our study, so it is unclear whether these indicators have guiding significance for the prognosis of patients, which is also one of the epidemiological research fields that must be improved.

## 5 Conclusions

Patients with SCCO have a poorer prognosis than patients with HGSOC. Older age at diagnosis, advanced disease stage, surgery status, radiation therapy, chemotherapy, number of primary tumors, larger tumor size, and bilateral tumor are the independent risk factors for poor survival of SCCO. The predictive models and nomograms were built to predict the individual survival rates of patients with SCCO or HGSOC, which has a promotion value for the clinic.

## Data availability statement

The original contributions presented in the study are included in the article/supplementary material. Further inquiries can be directed to the corresponding author.

## Author contributions

Conceptualization, DH and XL; methodology, DM; software, ZZ; validation, XL, DH, and DM; formal analysis, ZZ; investigation, YZ and KH; data curation, DM; writing—original draft preparation, XL; writing—review and editing, DH and DM; visualization, XL and DM; supervision, YZ; project administration, DM and ZZ; funding acquisition, DH, DM, and XL. All authors contributed to the article and approved the submitted version.
